# Subjective Straylight Index: A Visual Test for Retinal Contrast Assessment as a Function of Veiling Glare

**DOI:** 10.3390/jimaging10040089

**Published:** 2024-04-10

**Authors:** Francisco J. Ávila, Pilar Casado, Mª Concepción Marcellán, Laura Remón, Jorge Ares, Mª Victoria Collados, Sofía Otín

**Affiliations:** Departamento de Física Aplicada, Facultad de Ciencias, Universidad de Zaragoza, 50009 Zaragoza, Spainlauremar@unizar.es (L.R.); fatxutxa@unizar.es (J.A.);

**Keywords:** ocular straylight, image processing, glare spread function, straylight index, visual acuity, recovery time, ocular aberrations

## Abstract

Spatial aspects of visual performance are usually evaluated through visual acuity charts and contrast sensitivity (CS) tests. CS tests are generated by vanishing the contrast level of the visual charts. However, the quality of retinal images can be affected by both ocular aberrations and scattering effects and none of those factors are incorporated as parameters in visual tests in clinical practice. We propose a new computational methodology to generate visual acuity charts affected by ocular scattering effects. The generation of glare effects on the visual tests is reached by combining an ocular straylight meter methodology with the Commission Internationale de l’Eclairage’s (CIE) general disability glare formula. A new function for retinal contrast assessment is proposed, the subjective straylight function (SSF), which provides the maximum tolerance to the perception of straylight in an observed visual acuity test. Once the SSF is obtained, the subjective straylight index (SSI) is defined as the area under the SSF curve. Results report the normal values of the SSI in a population of 30 young healthy subjects (19 ± 1 years old), a peak centered at SSI = 0.46 of a normal distribution was found. SSI was also evaluated as a function of both spatial and temporal aspects of vision. Ocular wavefront measures revealed a statistical correlation of the SSI with defocus and trefoil terms. In addition, the time recovery (TR) after induced total disability glare and the SSI were related; in particular, the higher the RT, the greater the SSI value for high- and mid-contrast levels of the visual test. No relationships were found for low contrast visual targets. To conclude, a new computational method for retinal contrast assessment as a function of ocular straylight was proposed as a complementary subjective test for visual function performance.

## 1. Introduction

Visual perception is a cognitive process that starts with photoreception at the sensitive retina that converts the electromagnetic radiation into electro-chemical impulses. Those electric signals are received by the visual primary cortex for cognitive processing. Then, the beginning of visual perception lies on the neural encoding of the retinal image by the photoreceptor cells. Perception in visual function is driven by a contrast-based pathway [1].

The light entering the natural pupil of the eye is focused on the retina after refraction through the ocular media (i.e., the cornea, crystalline lens, aqueous and vitreous humors). Then, visual performance depends primarily on the retinal image contrast and resolution of the spatial frequency of the observed world [2]. 

The importance of retinal image contrast and the resolution of a wide range of spatial frequencies is critical in eye growth and cognitive development in children [3,4]. 

The operation principle of CS tests is based on contrast modulation of grating patterns for different spatial frequencies [5]; then, the contrast sensitivity function (CSF) assesses the ability to visualize a spatial pattern as a function of its contrast and size [6] and provides a powerful tool for discriminating visual impairments due to Parkinson disease [7], Alzheimer [8] or optic neuritis [9]. In addition, the CSF shape can characterize patients with glaucoma, cataract and age-related macular degeneration [10]. Estimating the CSF is a time-consuming subjective clinical procedure that has been speed up over the last years by implementing new CS test in tablet devices [6], computerized charts [11] and computer-adaptive tools [12]. Whereas CS tests are based on the threshold measure of contrast-modulated spatial gratings, contrast modulation procedures do not involve those main physical factors degrading the retinal image quality.

Retinal image quality can be affected by diffraction, ocular aberrations and scattering effects [13,14]. Whereas optical aberrations are mainly related to central vision, ocular scattering can affect the whole visual field. In particular, forward ocular scattering gives rise to the phenomenon of disability glare [15]. This concept lies in the dispersion that the light entering the eye suffers towards the retina due to particles and/or inhomogeneities of the ocular medium that gives rise to a veil of light that covers the whole retinal field. Superimposed veiling luminance causes a loss of retinal contrast that compromises visual performance and is known as glare [16]. Glare can be understood as the reduction in CS and the loss of visual acuity as a result of the scattering or straylight throughout the structures of the eye. The main straylight sources that affect vision are the ocular media themselves (i.e., tear film, iris pigmentation, cornea, sclera, crystalline lens and the retina) [17], aging [18], pathological conditions or external conditions such as the presence of bright light sources that interfere with the field of vision [19].

Depending on the generated straylight (which in turn depends on the brightness of the inrush light source and the ocular media itself), glare vision can be classified in two main groups: discomfort and disability glare [20]. According to the International Commission on Illumination (CIE), discomfort glare is defined as “glare that causes discomfort without necessarily impairing the vision of objects” [21]. On the contrary, disability glare impairs normal vision [22] without needing to cause discomfort glare.

In the last decade, the double-pass (DP) technology [23] has been applied to wide-field optical instruments and has allowed the reconstruction of the point spread function (PSF) of the human eye at larger angles than traditional DP systems that provide the PSF of the visual field (<1°) mainly affected by ocular aberrations [24].

Visual disability glare assessment should consider not only the objective measure of the glare covering the retinal field but also the neural processing of the retinal image affected by veiling glare (i.e., final visual perception). Over the past 50 years, many glare tests and straylight meters have been developed under a psychophysical methodology such as the Miller-Nadler glare instrument [25], Berkeley Glare Tester [26], brightness acuity tester [27] or Vistech MTC-8000 [28].

Although there seems to be no well-stablished standard instrument in clinical practice yet, the “C-Quant (Oculus, GmbH, Wetzlar, Germany)” device based on the compensation comparison method [29] is the most studied in the literature and is commercially available.

The aim of this work was to develop a complementary computational methodology to evaluate the subjective perception of straylight without the need for any optical instrument. The glare spread function (GSF) is employed to generate a bidimensional kernel for image convolution processing with visual acuity charts and then to generate visual tests affected by veiling glare. A new metric is introduced for the subjective visual evaluation of the spatial resolution affected by ocular straylight.

The subjective straylight function (SSF) evaluates the maximum tolerated straylight affecting visual testing based on the subject’s visual acuity. From the *SSF*, a new parameter defined as the subjective straylight index (SSI) is tested, establishing the average value in a sample of 30 young healthy volunteers. SSI is also correlated with ocular wavefront measures and temporal aspects of vision. The results show a relationship between *SSI* and high-order aberrations and with recovery time (RT) after inducing retinal photostress.

## 2. Materials and Methods

### 2.1. Computational Straylight Generator: The Glare Spread Function

Straylight can be measured by the equivalence luminance generated by an external source that causes the same visual veiling luminance perception as the bright disabling source at a given angular distance, *θ* [29]. Physically, straylight can be defined as the outer angular region (>1° outside the center) of the optical aberrations domain of the point spread function (PSF) [30] and can be quantified by the straylight parameter, *s* [31]: *s* = *θ*^2^·PSF.

**Figure 1:**
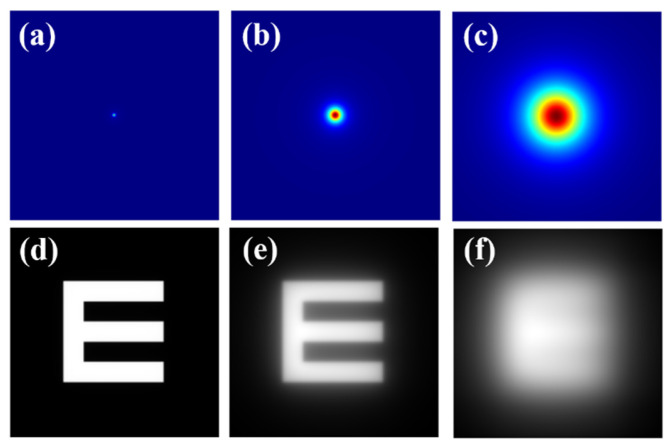
Top row: (**a**) diffraction-limited PSF (i.e., no straylight contribution); (**b**) *GSF* for *θ* = 4°; (**c**) *GSF* for *θ* = 8°. Bottom row: retinal images simulated for the PSF and GSFs shown at the upper row (**d**–**f**).

In the reported literature, *s* is specified at the glare angle *θ* at which the straylight is measured and given on a logarithmic scale with normal values around log (s) = 0.9 [32,33]. The *CIE* adopted the standard glare observer given by the total glare equation proposed by Vos and van den Berg [31] as:

(1) $$GSF\left(\theta \right)=\left[1-0.08*{\left(\frac{Age}{70}\right)}^{4}\right]*\left[\frac{9.2*10^{6}}{{\left[1+{\left(\frac{\theta }{0.046}\right)}^{2}\right]}^{1.5}}+\frac{1.5*10^{5}}{{\left[1+{\left(\frac{\theta }{0.045}\right)}^{2}\right]}^{1.5}}\right]+\left[1+1.6*{\left(\frac{Age}{70}\right)}^{4}\right]*\;\left\{\left[\frac{400}{1+{\left(\frac{\theta }{0.1}\right)}^{2}}+3*10^{-8}*\theta^{2}\right]+PF\left[\frac{1300}{{\left[1+{\left(\frac{\theta }{0.1}\right)}^{2}\right]}^{1.5}}+\frac{0.8}{{\left[1+{\left(\frac{\theta }{0.1}\right)}^{2}\right]}^{0.5}}\right]\right\}+2.5*10^{-3}*PF[{Sr}^{-1}]$$

where Age is the age of the observer and PF the eye pigment factor. The glare spread function (GSF) can be easily computed as a bidimensional kernel from Equation (1) as reported in [34]. The top row of Figure 1 shows computer-generated GSFs for different θ values. Then, any digital image (*i*) with known spatial characteristics can be characterized by its straylight (*i_s_*) through a mathematical convolutional processing given by:

(2) $$i_{s}=i⊗GSF(\theta )$$

As an example, the bottom row of Figure 1 shows an E-Snellen chart free of straylight (Figure 1d), and the same test affected by two different *θ* values of the GSF operator. GSF generation (Figure 1 top row) and image simulation (Figure 1 bottom row) were processed using a custom script written in Matlab 2019b (the MathWorks Inc., Natick, MA, USA) (a detailed description of the bidimensional GSF generation can be found elsewhere [34]).

### 2.2. Computational Straylight Detector: Straylight Index

**Figure 2:**
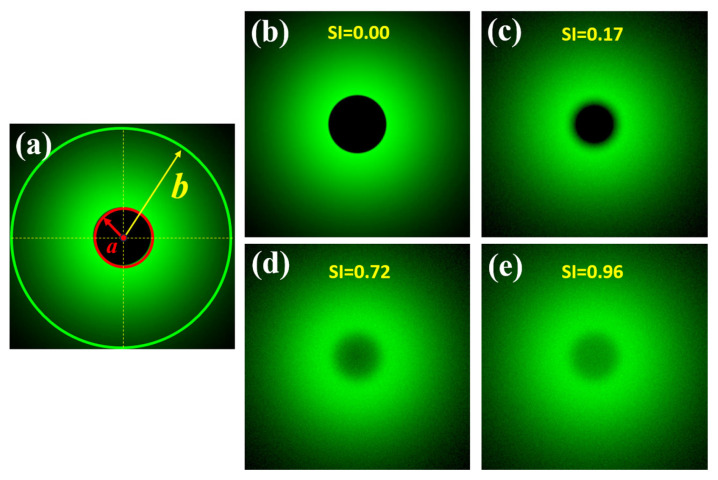
Scheme of the SI principle (**a**); simulated retinal images affected by different *SI* values (**b**–**e**). Retinal images were simulated in a straylight eye model (reported in [35]) using Zemax 13 optical design software (Zemax OpticStudio, LCC, Arlington Capital Partners, Washington, DC, USA).

A previous work published by our group reported a direct measurement of the wide-angle ocular straylight from retinal images [35]. The principle of operation is summarized as follows: The retina is illuminated by an extended bright annular ring that subtends 22° of the retinal field (*b*), the central part of the ring (*a* = 1.6° angular field) blocks the light acting as the measurement area (See Figure 2a). Figure 2b shows a retinal image from an artificial eye without any straylight effect. If a straylight source (corneal edema, cataracts or an external bright source, for instance) characterizes the PSF of the eye with intraocular diffusion, as shown in Figure 1, the retinal image of Figure 2b is covered by a uniform veil of light called veiling glare (Figure 1c–e), as a consequence of the increased scattered light throughout the ocular medium.

Then, the straylight index (SI) parameter can be computed for a given visual angle α, as follows [35]:

(3) $$SI=\frac{\int_{0}^{a}I(\alpha )\cdot d\alpha }{\int_{0}^{b}I(\alpha )\cdot d\alpha }$$

### 2.3. Straylight Index as a Measure of the Glare Spread Function

**Figure 3:**
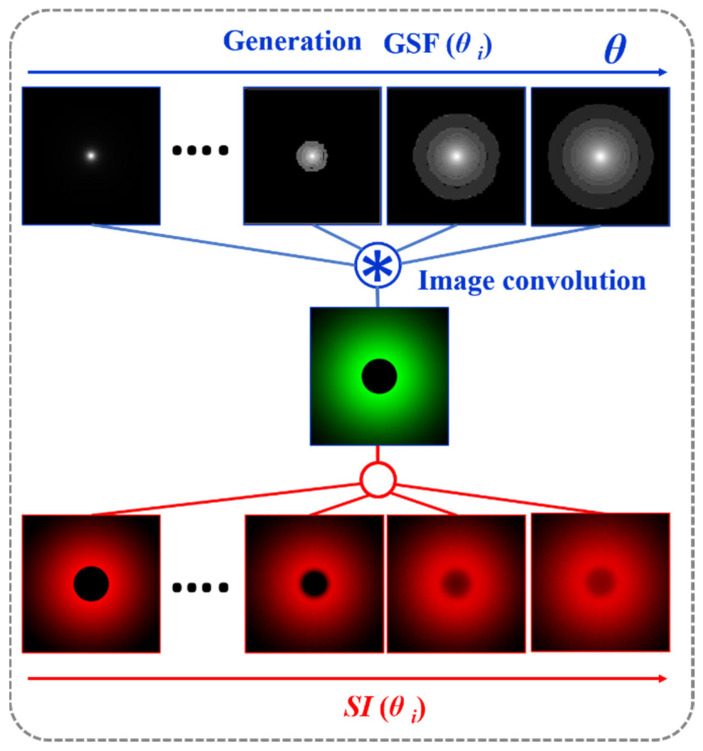
Convolutional image processing to obtain the SI parameter as a function of the glare angle (θ) of the glare spread function.

**Figure 4:**
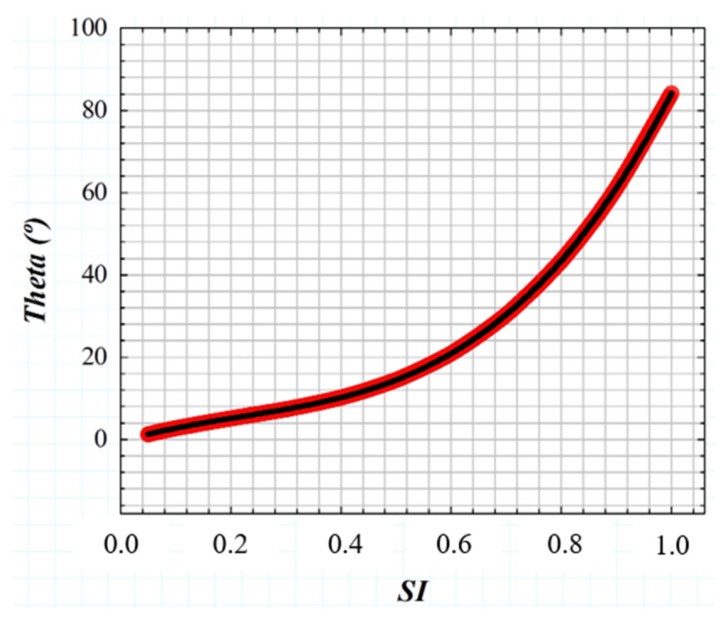
Numerical results of SI values obtained from computer generated retinal images affected by straylight (i.e., characterized by the GSF (θ)).

If the *GSF* (Equation (1)) as a function of the glare angle (θ) and the working principle of the wide-field straylight meter [36] (Figure 2a) are combined into a convolution procedure shown in Figure 3, any retinal image affected by the GSF (θ) can be characterized by a numeric SI value. Figure 4 shows the computed straylight index (SI) values in simulated retinal images affected by GSF as a function of the glare angle (θ). The numerical results shown in Figure 4 can be fitted by the following expression:

(4) $$\theta ={A\cdot {SI}^{3}+B\cdot {SI}^{2}+C\cdot SI+D}^{\;}$$

where A = 130.25, B = −85.92, C = 40.46 and D = −0.58.

**Figure 5:**
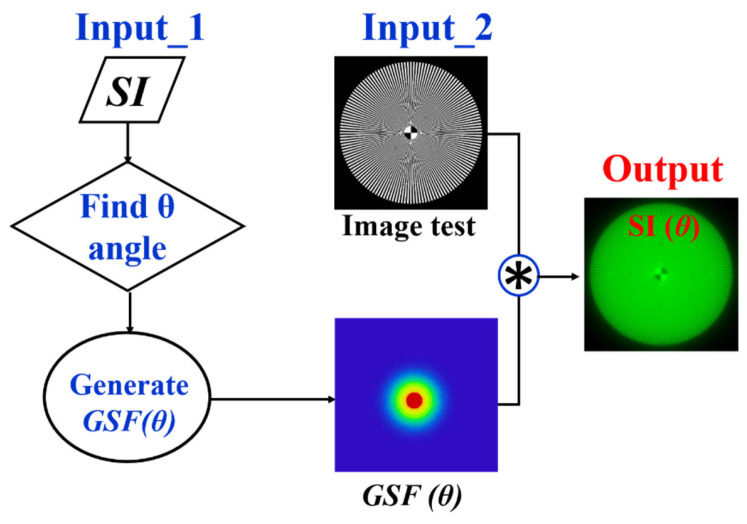
Image processing procedure to generate veiling glare in any digital image quantified by SI (θ).

Given the relationship between the SI parameter and the glare angle (θ) of the GSF, any visual chart can be processed to be affected by straylight as shown in Figure 5. First, an SI value is chosen and its corresponding θ is found using the Equation (3). The test image size is then read out to generate a bidimensional kernel GSF with the same spatial resolution. Finally, a convolutional image processing returns the test image affected by straylight.

**Figure 6:**
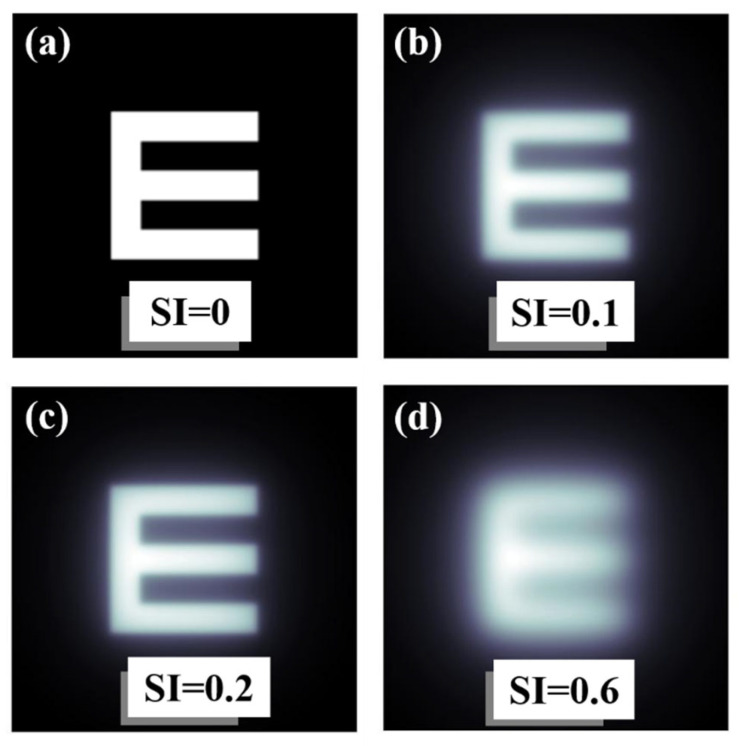
Examples of an E-letter optotype affected by different values of SI. Straylight-free (**a**) and optotypes affected by SI = 0.1 (**b**), SI = 0.2 (**c**) and SI = 0.6 (**d**) values.

As an example of operation, Figure 6 shows computational results obtained from the convolutional procedure described in Figure 3. Figure 6a shows a Snellen E-letter optotype and the same image affected by different *SI* values (Figure 6b–d). Veiling glare effects are easily visible as the SI increases.

### 2.4. Subjective Straylight Function

**Figure 7:**
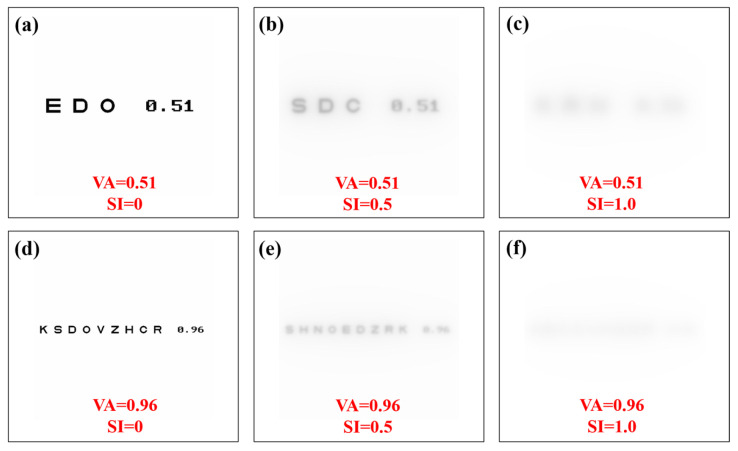
Visual acuity charts for different values of visual acuity and straylight index. Straylight-free VA charts (**a**,**d**) and for SI = 0.5 (**b**,**e**) and SI = 1.0 (**c**,**f**) values.

A Sloan visual acuity chart optotype script [36] was modified to generate visual acuity (VA) charts ranging from 0.3 to 1.2 (decimal notation). For each VA chart generated, image convolution processing was performed to generate VA optotypes affected by straylight. Then, a total of 10 convolutional processes were computed for each VA value (i.e., 110 VA optotypes as a function of the SI value, all files are available in Supplementary Materials). Figure 7 shows examples of generated optotypes for different values of VA and SI. 

The subjective straylight function (SSF) is given by the maximum tolerated SI value for each VA Sloan chart. The subjective straylight index (SSI) is then defined as the area under the SSF. The larger the area under the SSF, the better the tolerance of the visual system to preserve contrast sensitivity when ocular straylight veils the retinal field. SSI can be computed as:

(5) $$SSI=\int_{{VA}_{min}}^{{VA}_{max}}SSF\cdot d(VA)$$

Here, VA_min_ = 0.3 and VA_max_ = 1.2.

### 2.5. Subjects

Thirty young adult subjects (19 ± 1 years old) participated in this study. Participants with ocular pathologies, visual impairment or uncorrected refractive errors were excluded from the sample. All of them were asked to use their optometric correction (i.e., wear contact lenses or spectacles) during the visual test.

### 2.6. Wavefront Aberrometry

Ocular aberrometry was measured using a laser ray tracing system (iTrace, Tracey Technologies, Houston, TX, USA). A total of 24 Zernike terms were calculated for a pupil size of 6 mm (diameter). Root-mean-square (RMS) values for total low- and high-order aberrations (LOA and HOA, respectively) as well as defocus, spherical aberration, coma and trefoil terms were obtained monocularly (right eye) for each subject. 

### 2.7. Time Recovery from Total Disability Glare

**Figure 8:**
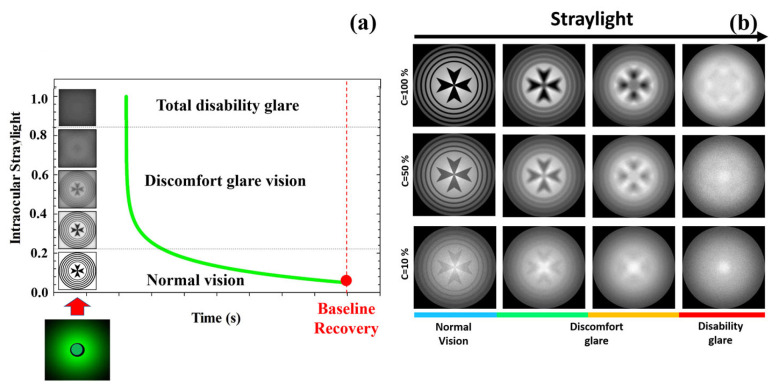
Temporal phases of disability glare vision after flash-lighting until baseline vision is recovered (**a**); simulated retinal images as a function of the contrast of the visual target and ocular straylight (**b**). Blue bar corresponds to normal vision; green and orange to medium and high straylight in discomfort glare regime and finally red bar corresponds to a severe degree of straylight in disability glare vision.

An optical disability glare instrument [37] was employed to measure the recovery time after retinal photostress for different levels of Michelson contrast of the visual target: 100%, 50%, 25%, 10% and 5%. Briefly, the experimental procedure consisted in perceiving (monocularly) the visual test with the given contrast sensitivity, and then total disability glare (i.e., retinal photobleaching) was induced by triggering a bright glare source during a short exposure time of 240 milliseconds (a detailed description of the instrument and measurements’ protocol can be found elsewhere [37]). As mentioned in the Introduction, disability glare can range between two main stages from normal vision: discomfort glare and total disability glare. Figure 8a depicts the time course of disability glare after retinal photostress induced by a dazzling light. At the moment of flashing, the straylight causes retinal veiling glare which makes it impossible to discriminate any spatial frequency pattern (i.e., temporal blindness). Before recovering normal vision (baseline), the visual system goes through the intermediate regime between total disability glare and normal vision: discomfort glare. The time required to return to baseline (recovery time, RT) is the clinical parameter used by the disability glare instrument. For a sense of completeness, Figure 8b shows retinal image simulations for a visual target display at different levels of Michelson contrast and how contrast sensitivity degrades as straylight increases. Retinal images were generated by optical simulation on a previously reported straylight eye model [35].

### 2.8. Image Processing, Data Analysis and Statistics

*GSF* kernel generation image convolution and *VA* optotypes’ algorithms for the *SSF* and *SSI* evaluation (see available files in Supplementary Materials) were custom-programmed using Matlab2019b (the MathWorks Inc., Natick, MA, USA). The statistical analysis consisted of the Pearson correlation coefficient to establish significant correlation between the *SSI* parameter and the data of the aberrometric and visual temporal aspects. Graphical representation and statistics were performed in Origin Lab software (OriginPro 2024, Origin Lab Corp., Northampton, MA, USA). Optical simulations were implemented in Zemax optical design software (Zemax OpticStudio, LCC, Arlington Capital Partners, Washington, DC, USA).

## 3. Results

### 3.1. Subjective Straylight Index in Young Healthy Subjects

**Figure 9:**
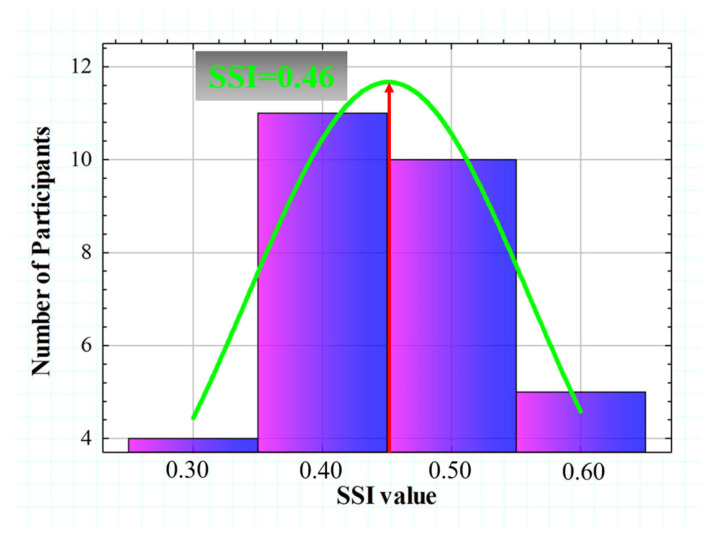
Histogram representation of the *SSI* value for all participants. A gaussian fit (R^2^ = 0.82) was found with a peak centered at SSI = 0.46.

SSI values calculated from the 30 subjects ranged from 0.365 to 0.680. Figure 9 shows the histogram representation of the SSI (for four intervals), which is normally distributed with an asymmetric gaussian shape (R^2^ = 0.82). The gaussian peak was found at a value of SSI = 0.46.

**Figure 10:**
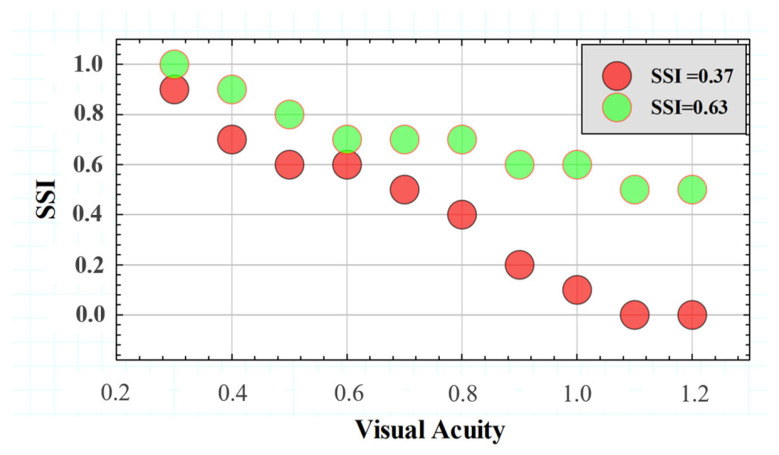
*SSFs* for two subjects with different *SSI* values.

Figure 10 compares the SSF for two different subjects with extreme SSI values of the Gaussian distribution shown in Figure 10 above. The SSF curves showed similar behavior for the range between VA values of 0.3 and 0.6. However, starting from a VA higher than 0.6, the SSI values decreased drastically, while the subject with SSI = 0.63 remained with a relatively constant tolerance to induced straylight in the optotypes shown. According to the data, the higher the SSI value, the greater the area under the SSF curve, which may translate into the visual system’s ability to preserve contrast sensitivity in the presence of external straylight sources.

### 3.2. Effect of SSI on the Spatial Resolution of the Eye: Ocular Wavefront

The influence of straylight on spatial vision is well reported, and as a relevant aspect, its capability as a predictor of visual performance in night-driving conditions [38] or as an indicator of cataract surgery [39]. This section studies how the SSI can be affected by ocular aberrations and then the influence of the eye optics on the final visual perception of veiling glare. Table 1 shows the averaged root-mean-square (RMS) values for low- and high-order aberrations and for the specific low-order defocus and high-order coma, spherical aberration and trefoil terms.

**Figure 11:**
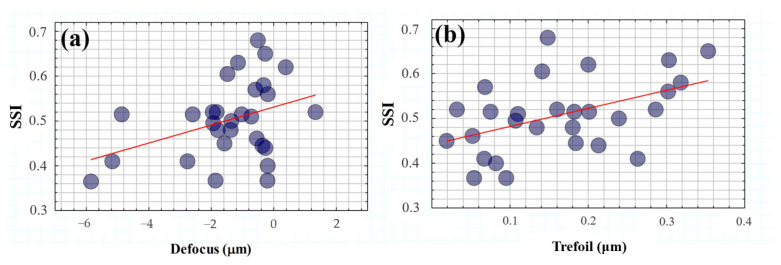
*SSI* as a function of defocus (**a**) and trefoil (**b**) terms. The red lines correspond to the linear statistical correlations of *SSI* with both defocus (R^2^ = 0.39, *p* = 0.03) and trefoil (R^2^ = 0.46, *p* = 0.012) terms.

Figure 11 shows the SSI values as a function of low (Figure 11a) and high-order terms (Figure 11b). A statistical analysis revealed a significant dependence of the SSI parameter on defocus (R^2^ = 0.39, *p* = 0.03) and trefoil (R^2^ = 0.46, *p* = 0.012).

On the one hand, these results corroborate the evident impact of optical blur on visual spatial resolution but under the influence of glare effects. In addition, the modulation of ocular trefoil appears to play an important role in visual tolerance to straylight.

### 3.3. Effect of SSI on Visual Contrast Sensitivity Recovery Time

**Figure 12:**
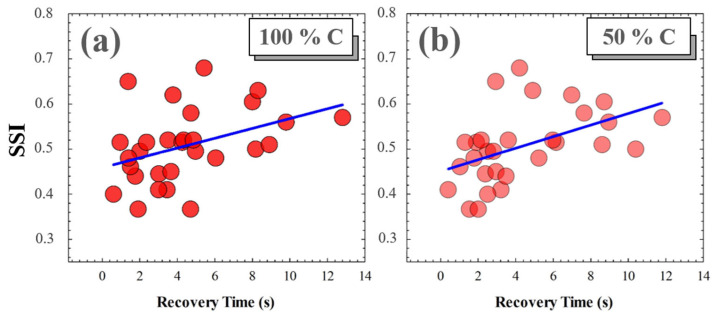
SSI as a function of RT for 100% (**a**) and for 50% visual test contrast levels (**b**). Blue lines correspond to linear statistical correlations.

A photostress measure was carried out for each volunteer as a function of the Michelson contrast of the visual target. No significant relationships were found between SSI and recovery time (RT) for low contrast levels (i.e., for 25%, 10% and 5% Michelson contrast values). However, for medium and high contrast levels, the *RT* from disability glare depended on the subject’s SSF. Figure 12 shows the correlations found between SSI and RT after total induced disability glare. The average RTs for 100% and 50% contrast levels were 4.41 and 4.38 s, respectively; these small differences were not statistically significant (*p* = 0.982).

## 4. Discussion and Conclusions

We developed a new methodology for computational modelling of ocular straylight in visual acuity tests using convolutional image processing. The CIE disability glare formula [31] and direct straylight meter methodology [35] were combined to generate and measure the effects of veiling glare on visual perception. The generation of straylight in visual tests was performed by convolving the images (veiling glare) with the glare spread function [34] based on the Straylight Index [35].

Visual acuity was assessed in 30 healthy young subjects as a function of the induced SI. The subjective measure of the maximum resolved SI value for each value of VA provides the Subjective Straylight Function (SSF) that addresses the neural and optical attenuation thresholds of VA due to ocular straylight effects. The area under the SSF curve is defined as the Subjective straylight index (SSI).

The SSI parameter was tested in a young population and showed a normal distribution with a peak centered at SSI = 0.46. Once the normal value was established, the influence of the spatial and temporal aspects of vision on SSI was analyzed.

Wavefront aberrometry and RT after induced total disability glare were measured and correlated with SSI. LOA played a role in SSF and in particular the SSI value was found to be negatively correlated with the defocus term, similar to the degradation found in edge contrast sensitivity (*CS*) due to optical blur [40].

Fernández-Sanchez et al. [41] investigated the effect of high-order aberrations on human vision; they found relationships between visual CS with coma and third-order trefoil terms. In particular, they found a significant reduction in CS with induced coma and trefoil values of around (or more than) 1.03 and 0.96 μm, respectively. In agreement with their results, our findings revealed a negative statistical correlation between SSI and trefoil values (mean values 0.18 ± 0.14 μm); however, no relationship was found between SSI and coma, probably due to the relatively low values and mean of our sample (0.25 ± 0.40 μm).

Finally, it has been reported that RT after induced total disability glare is significantly increased in myopic eyes [42]. Our finding revealed a significant correlation between RT and SSI values for high (100%) and medium (50%) Michelson contrast levels of visual targets. However, SSI was independent of RT for low levels of visual target contrast.

To conclude, a new computational visual test was proposed for the evaluation of retinal contrast based on ocular straylight. VA charts characterized by the straylight index allowed us to define the SSF and the SSI parameter as a neuro-optical measure of visual perception under glare vision conditions. The SSI depends not only on optical blur (defocus) but also on high-order aberrations (trefoil). Furthermore, higher SSI values are related to longer recovery times after retinal photostress for high and medium contrast levels.

Future work will include a clinical study in patients affected by ocular straylight sources and the incorporation into the visual computational method of a functionality for the modulation of ocular aberrations in combination with straylight effects.

## Figures and Tables

**Table 1:** RMS values for low (LOA) and high-order aberrations (HOA) and for defocus, coma, spherical aberration (spherical ab.) and trefoil. The asterisk marks those terms for which statistical correlations with *SSI* were found.

RMS LOA	RMS HOA	Defocus *	Coma	Spherical Ab.	Trefoil *
1.82 ± 1.50	0.44 ± 0.59	−1.29 ± 1.73	0.25 ± 0.40	0.01 ± 0.33	0.18 ± 0.14

## Data Availability

Visual charts are available in the Github repository https://github.com/favilago/SSI (accessed on 9 April 2024).

## Supplementary Materials

The visual acuity charts based on SI to obtain the SSF can be found in this repository: https://github.com/favilago/SSI (accessed on 9 April 2024).

## References

[1] Xiao N., Xu S., Li Z.-K., Tang M., Mao R., Yang T., Ma S.-X., Wang P.-H., Li M.-T., Sunilkumar A. (2023). A single photoreceptor splits perception and entrainment by cotransmission. Nature.

[2] Liu L., Wang Y., Liu J., Liu W. (2018). Retinal-image quality and contrast sensitivity function in eyes with epiretinal membrane: A cross-sectional observational clinical study. BMC Ophthalmol..

[3] Maurer D., Mondloch C.J., Lewis T. (2007). L Effects of early visual deprivation on perceptual and cognitive development. Prog. Brain Res..

[4] Tran N., Chiu S., Tian Y., Wildsoet C.F. (2008). The significance of retinal image contrast and spatial frequency composition for eye growth modulation in young chicks. Vis. Res..

[5] Jindra L.F., Zemon V. (1989). Contrast sensitivity testing: A more complete assessment of vision. J. Cataract. Refract. Surg..

[6] Dorr M., Lesmes L.A., Elze T., Wang H., Lu Z.L., Bex P.J. (2017). Evaluation of the precision of contrast sensitivity function assessment on a tablet device. Sci. Rep..

[7] Ming W., Palidis D.J., Spering M., McKeown M.J. (2016). Visual Contrast Sensitivity in Early-Stage Parkinson’s Disease. Investig. Ophthalmol. Vis. Sci..

[8] Cormack F.K., Tovee M., Ballard C. (2000). Contrast sensitivity and visual acuity in patients with Alzheimer’s disease. Int. J. Geriatr. Psychiatry.

[9] Mahayana I.T., Sakti D.H., Gani T.T. (2021). Automated grating contrast-sensitivity: The easy test for hidden visual loss in recovered optic neuritis patient. Taiwan. J. Ophthalmol..

[10] Kara S., Gencer B., Ersan I., Arikan S., Kocabiyik O., Tufan H.A., Comez A. (2016). Repeatability of contrast sensitivity testing in patients with age-related macular degeneration, glaucoma, and cataract. Arq. Bras. Oftalmol..

[11] Savini G., Calossi A., Schiano-Lomoriello D., Barboni P. (2019). Precision and Normative Values of a New Computerized Chart for Contrast Sensitivity Testing. Sci. Rep..

[12] Rosenkranz S.C., Kaulen B., Zimmermann H.G., Bittner A.K., Dorr M., Stellmann J.P. (2021). Validation of Computer-Adaptive Contrast Sensitivity as a Tool to Assess Visual Impairment in Multiple Sclerosis Patients. Front. Neurosci..

[13] Martínez-Roda J.A., Vilaseca M., Ondategui J.C., Giner A., Burgos F.J., Cardona G., Pujol J. (2011). Optical quality and intraocular scattering in a healthy young population. Clin. Exp. Optom..

[14] Liao X., Lin J., Tian J., Wen B., Tan Q., Lan C. (2018). Evaluation of optical quality: Ocular scattering and aberrations in eyes implanted with diffractive multifocal or Monofocal intraocular lenses. Curr. Eye Res..

[15] Donald R.B. (1981). Veiling glare reduction methods compared for ophthalmic applications. Appl. Opt..

[16] Van den Berg T.J.T.P., Franssen L., Kruijt B., Coppens J.E. (2013). History of ocular straylight measurement: A review. Med. Phys..

[17] Van den Berg T.J.T.P., IJspeert J.K., De Waard P.W. (1991). Dependence of intraocular straylight on pigmentation and light transmission through the ocular wall. Vis. Res..

[18] Mueller-Schotte S., Van der Schouw Y.T., Schuurmans M.J. (2015). Ocular Straylight: A Determinant of Quality of Life in the Elderly?. Gerontol. Geriatr. Med..

[19] Ayama M., Yamazaki R., Nakanoya S.I., Tashiro T., Ishikawa T., Ohnuma K., Shinoda H., Araki K. (2015). Estimation of straylight in the eye and its relation to visual function. Opt. Rev..

[20] Mainster M.A., Turner P.L. (2012). Glare’s causes, consequences, and clinical challenges after a century of ophthalmic study. Am. J. Ophthalmol..

[21] Commission Internationale de l’Éclairage (2019). CIE e-ILV Term 17-333 Discomfort Glare.

[22] Commission Internationale de l’Éclairage (2019). CIE e-ILV Term 17-492 Glare.

[23] Galliot F., Patel S.R., Cochener B. (2016). Objective Scatter Index: Working Toward a New Quantification of Cataract?. J. Refract. Surg..

[24] Ginis H., Pérez G.M., Bueno J.M., Artal P. (2012). The wide-angle point spread function of the human eye reconstructed by a new optical method. J. Vis..

[25] Miller D., Jernigan M.E., Molnar S., Wolf E., Newman J. (1972). Laboratory evaluation of a clinical glare tester. Arch. Opthalmol.

[26] Bailey I.L., Bullimore M.A. (1991). A new test for the evaluation of disability glare. Optom. Vis. Sci..

[27] Holladay J.T., Prager T.C., Trujillo J., Ruiz R.S. (1987). Brightness acuity test and outdoor visual acuity in cataract patients. J. Cataract. Refract. Surg..

[28] Elliott D.B., Bullimore M.A. (1993). Assessing the reliability, discriminative ability, and validity of disability glare tests. Investig. Ophthalmol. Vis. Sci..

[29] Franssen L., Coppens J.E., Van den Berg T.J. (2006). Compensation comparison method for assessment of retinal straylight. Investig. Ophthalmol. Vis. Sci..

[30] Vos J.J., Van den Berg T.J.T.P. (1997). On the course of the disability glare function and its attribution to components of ocular scatter. CIE Collect..

[31] Vos J.J., Van den Berg T.J.T.P. (1999). Report on disability glare. CIE Collect..

[32] Van den Berg T.J.T.P. (1995). Analysis of intraocular straylight, especially in relation to age. Optom. Vis. Sci..

[33] Van Den Berg T.J., Van Rijn L.R., Michael R., Heine C., Coeckelbergh T., Nischler C., Wilhelm H., Grabner G., Emesz M., Barraquer R.I. (2007). Straylight effects with aging and lens extraction. Am. J. Ophthalmol..

[34] Ávila F.J., Ares J., Marcellán M.C., Collados M.V., Remón L. (2021). Iterative-Trained Semi-Blind Deconvolution Algorithm to Compensate Straylight in Retinal Images. J. Imaging.

[35] Ávila F., Collados M.V., Ares J., Remón L. (2020). Wide-field direct ocular straylight meter. Opt. Express.

[36] SergioBonaqueGonzalez/Sloan-Visual-Acuity-Eye-Chart-Optotype. https://github.com/SergioBonaqueGonzalez/Sloan-Visual-Acuity-Eye-Chart-Optotype.

[37] Ávila F.J., Casado P. (2022). Optical instrument for the study of time recovery from total disability glare vision. Appl. Opt..

[38] Ungewiss J., Schiefer U., Eichinger P., Wörner M., Crabb D.P., Jones P.R. (2022). Does intraocular straylight predict night driving visual performance? Correlations between straylight levels and contrast sensitivity, halo size, and hazard recognition distance with and without glare. Front. Hum. Neurosci..

[39] Van Bree M.C.J., Pierrache L., Zijlmans B.L.M., Reus N.J., Van den Born L.I., Van den Berg T.J.T.P. (2017). Straylight as an Indicator for Cataract Extraction in Patients with Retinal Dystrophy. Ophthalmol. Retin..

[40] Jansonius N.M., Kooijman A.C. (1997). The effect of defocus on edge contrast sensitivity. Ophthalmic Physiol. Opt..

[41] Fernández-Sánchez V., Ponce M.E., Lara F., Montés-Micó R., Castejón-Mochón J.F., López-Gil N. (2008). Effect of 3rd-order aberrations on human vision. J. Cataract. Refract. Surg..

[42] Ávila F.J., Casado P., Ares J. (2023). Photostress Recovery Time after Flash-Lighting Is Increased in Myopic Eyes. Photonics.

